# First comprehensive proteome analysis of lysine crotonylation in *Streptococcus agalactiae*, a pathogen causing meningoencephalitis in teleosts

**DOI:** 10.1186/s12953-021-00182-y

**Published:** 2021-11-10

**Authors:** Xinjin Chen, Bolin Fan, Chenlong Fan, Zhongliang Wang, Eakapol Wangkahart, Yucong Huang, Yu Huang, Jichang Jian, Bei Wang

**Affiliations:** 1 Guangdong Provincial Key Laboratory of Pathogenic Biology and Epidemiology for Aquatic Economic Animal, Key Laboratory of Control for Disease of Aquatic Animals of Guangdong Higher Education Institutes, College of Fishery, Guangdong Ocean University, Southern Marine Science and Engineering Guangdong Laboratory (Zhanjiang), Zhanjiang, PR China; 2 Key Laboratory of Experimental Marine Biology, Institute of Oceanology, Chinese Academy of Sciences, Qingdao, China Laboratory for Marine Biology and Biotechnology, Qingdao National Laboratory for Marine Science and Technology, Qingdao, China; 3 Research Unit of Excellence for Tropical Fisheries and Technology, Division of Fisheries, Department of Agricultural Technology, Faculty of Technology, Mahasarakham University, Khamriang Sub-District, Kantarawichai, Mahasarakham Thailand

**Keywords:** *Streptococcus agalactiae*, Crotonylation, Virulence factors, Proteomics

## Abstract

**Backgroud:**

*Streptococcus agalactiae* is a common colonizer of the rectovaginal tract and lead to infectious diseases of neonatal and non-pregnant adults, which also causes infectious disease in fish and a zoonotic risk as well. Lysine crotonylation (Kcr) is a kind of histone post-translational modifications discovered in 2011. In yeast and mammals, Kcr function as potential enhancers and promote gene expression. However, lysine crotonylation in *S. agalactiae* has not been studied yet.

**Methods:**

In this study, the crotonylation profiling of fish pathogen, *S. agalactiae* was investigated by combining affinity enrichment with LC MS/MS. The Kcr modification of several selected proteins were further validated by Western blotting.

**Results:**

In the present study, we conducted the proteome-wide profiling of Kcr in *S. agalactiae* and identified 241 Kcr sites from 675 screened proteins for the first time. Bioinformatics analysis showed that 164 sequences were matched to a total of six definitively conserved motifs, and many of them were significantly enriched in metabolic processes, cellular process, and single-organism processes. Moreover, four crotonylation modified proteins were predicted as virulence factors or to being part of the quorum sensing system PTMs on bacteria. The data are available via ProteomeXchange with identifier PXD026445.

**Conclusions:**

These data provide a promising starting point for further functional research of crotonylation in bacterial virulence in *S. agalactiae*.

**Supplementary Information:**

The online version contains supplementary material available at 10.1186/s12953-021-00182-y.

## Background

Lysine crotonylation is a newly discovered post-translational modification, which is structurally and functionally different from the widely studied lysine acetylation [1]. Crotonylation occurs on the ε-amino group of lysine but differ from acetylation in its four-carbon length and planar orientation [2]. In order to characterize the global crotonylation proteome, a proteomic method based on sensitive immune-affinity purification and high-resolution liquid chromatography-tandem mass spectrometry (LC-MS/MS) was applied to identify new crotonylated proteins and modification sites. With the utilization of high specificity antibodies, increasing crotonylated lysine residues and crotonylated proteins were identified so far. Also, the involvement of crotonylated proteins in multiple cellular processes were well-recorded [3].

In bacterial cells, lysine acylation (Kace) modification has been well studied in many species, including *Vibrio alginolyticus*, *Vibrio parahaemolyticus*, *Escherichia coli* and *Mycobacterium tuberculosis* [4–7]. The Kace modification was involved in many basal physiological functions such as chemotaxis, virulence, and antibiotic resistance [8–10]. However, the crotonylome profile of bacterial pathogen of fish is still unclear. *Streptococcus agalactiae* or group B streptococcus (GBS) is an important bacterial in fish that result in abnormal swimming behaviour, popeye, haemorrhages, and severe mortalities. GBS is also an important zoonotic pathogen due to the fact that it can infect human as well [11]. Recently, several proteins of GBS such as LuxS [12] and RbsB [13] participated in AI-2-mediated quorum sensing system. However, the intrinsic quorum sensing mechanisms are still largely unknown, especially the roles of Kac modification.

In present study, we investigated the global lysine crotonylation proteome of *S. agalactiae* using high-resolution LC-MS/MS coupled with highly sensitive immune-affinity purification. We conducted proteome-wide profiling of Kcr in *S. agalactiae* and identified 241 Kcr sites from 675 proteins, indicating the Kcr exist in *S. agalactiae*. Bioinformatics analysis showed that 164 sequences were matched to a total of six definitively conserved motifs, and many of them were significantly enriched in metabolic processes, cellular process, and single-organism processes. Moreover, four contonylation modified proteins was associated with bacterial QS system and virulence. Our data expand the understanding of Kcr modification profiling in prokaryotes and provides new insights into the potential role of PTMs in fish pathogens.

## Methods

### Bacterial materials and growth conditions

S*.agalactiae* ZQ0910, a virulent strain isolated from intensive tilapia farm with a typical streptococcicosis outbreak in the Guangdong province of China in 2009 [14]. It belongs to serotype Ia and used for challenge study. The strain was grown aerobically overnight at 28 °C in a shaker bath, and then overnight cultured cells were diluted into 1:100 in BHI medium. Bacteria were then harvested by centrifugation (5000 g, 5 min). Cell pellets were washed and resuspended in PBS to obtain the desired concentrations for the infection doses. The final bacterial concentrations were confirmed by plating ten-fold serial dilutions onto blood-agar plates.

### Protein extraction

Sample was sonicated three times on ice using a high intensity ultrasonic processor (Scientz) in lysis buffer (8 M urea, 3 μM TSA and 50 mM NAM and 1% Protease Inhibitor Cocktail, Millipore). The remaining debris was removed by centrifugation at 12,000 g at 4 °C for 10 min. Finally, the supernatant was collected and the protein concentration was determined with BCA kit according to the manufacturer’s instructions.

### Trypsin digestion

For digestion, the protein solution was reduced with 5 mM dithiothreitol (final concentration) for 30 min at 56 °C and alkylated with 11 mM iodoacetamide (final concentration) for 45 min at room temperature in darkness. The protein sample was then diluted by adding 100 mM TEAB to urea concentration less than 2 M. Finally, trypsin was added at 1:50 trypsin-to-protein mass ratio for the first digestion overnight and 1:100 trypsin-to-protein mass ratio for a second 4 h-digestion [15].

### Affinity enrichment

To enrich Kcro peptides, tryptic peptides dissolved in NETN buffer (100 mM NaCl, 1 mM EDTA, 50 mM Tris-HCl, 0.5% NP-40, pH 8.0) were incubated with pre-washed antibody beads (PTM Biolabs) at 4 °C overnight with gentle shaking. Then the beads were washed four times with NETN buffer and twice with H_2_O. The bound peptides were eluted from the beads with 0.1% trifluoroacetic acid. Finally, the eluted fractions were combined and vacuum-dried. For LC-MS/MS analysis, the resulting peptides were desalted with C18 ZipTips (Millipore) according to the manufacturer’s instructions, followed by LC-MS/MS analysis.

### Quantitative proteomic analysis by LC-MS/MS

The tryptic peptides were dissolved in 0.1% formic acid (solvent A), directly loaded onto a reversed-phase pre-column (Acclaim PepMap 100; Thermo Fisher Scientific, Inc.). Peptide separation was performed using a reversed-phase analytical column (Acclaim PepMap RSLC; Thermo Fisher Scientific, Inc.) with gradient that comprised of an increase from 6 to 23% solvent B (0.1% formic acid in 98% acetonitrile) over 26 min, 23 to 35% in 8 min and climbing to 80% in 3 min then holding at 80% for the last 3 min, all at a constant flow rate of 400 nL/min on an EASY-nLC 1000 UPLC system [16].

The peptides were subjected to nanospray ion source (NSI) source followed by tandem mass spectrometry (MS/MS) in Q ExactiveTM Plus (Thermo) coupled online to the UPLC. The electrospray voltage applied was 2.0 kV. The m/z scan range was 350 to 1800 for full scan, and intact peptides were detected in the Orbitrap at a resolution of 70,000. Peptides were then selected for MS/MS using normalized collision energy (NCE) setting as 28 and the fragments were detected in the Orbitrap at a resolution of 17,500. A data-dependent procedure that alternated between one MS scan followed by 20 MS/MS scans with 15.0 s dynamic exclusion. The electrospray voltage applied was 2.0 kV. Automatic gain control (AGC) was used to prevent overfilling of the Qrbitrap; 50,000 ions were accunulated to generate MS/MS spectra. For MS scans, the m/z scan range was 350 to 1800. The fixed first mass was set as 100 m/z.

### Database search

The resulting MS/MS data were processed using Maxquant search engine (v.1.5.2.8). Tandem mass spectra were searched against 1123 database concatenated with reverse decoy database. Trypsin/P was specified as cleavage enzyme allowing up to 4 missing cleavages. The mass tolerance for precursor ions was set as 20 ppm in First search and 5 ppm in Main search, and the mass tolerance for fragment ions was set as 0.02 Da. Carbamidomethyl on Cys was specified as fixed modification and crotonylation on lysine were specified as variable modifications. FDR was adjusted to < 1% and minimum score for modified peptides was set > 40. For selected specific Kcr sites, site localization probability was set to > 0.75. All other parameters in MaxQuant were used as default.

### Bioinformatics analysis for gene ontology annotation

The Gene Ontology, or GO, is a major bioinformatics initiative to unify the representation of gene and gene product attributes across all species. Gene Ontology (GO) annotation proteome was derived from the UniProt-GOA database (www. http://www.ebi.ac.uk/GOA/) [17]. Firstly, Converting identified protein ID to UniProt ID and then mapping to GO IDs by protein ID. If some identified proteins were not annotated by UniProt-GOA database, the InterProScan soft would be used to annotated protein’s GO functional based on protein sequence alignment method. Then proteins were classified by Gene Ontology annotation based on three categories: biological process, cellular component and molecular function.

For subcelluar localization, we used wolfpsort a subcellular localization predication soft to predict subcellular localization. Wolfpsort is an updated version of PSORT/PSORT II for the prediction of eukaryotic sequences. Special for protokaryon species, Subcellular localization prediction soft CELLO was used.

Kyoto Encyclopedia of Genes and Genomes (KEGG ) database was used to annotate protein pathway. Firstly, using KEGG online service tools KAAS to annotated protein’s KEGG database description. Then mapping the annotation result on the KEGG pathway database using KEGG online service tools KEGG mapper.

### Bioinformatics analysis for enrichment GO and KEGG pathway analysis

Proteins were classified by GO annotation into three categories: biological process, cellular compartment and molecular function. For each category, a two-tailed Fisher’s exact test was employed to test the enrichment of the identified modified protein against all proteins of the species database. The GO with a corrected *p*-value < 0.05 is considered significant.

Encyclopedia of Genes and Genomes (KEGG) database was used to identify enriched pathways by a two-tailed Fisher’s exact test to test the enrichment of the identified modified protein against all proteins of the species database. The pathway with a corrected *p*-value < 0.05 was considered significant. These pathways were classified into hierarchical categories according to the KEGG website.

### Motif analysis

Soft MoMo (motif-x algorithm) was used to analysis the model of sequences constituted with amino acids in specific positions of modify-21-mers (10 amino acids upstream and downstream of the site, but phosphorylation with modify-13-mers that 6 amino acids upstream and downstream of the site) in all protein sequences. And all the database protein sequences were used as background database parameter. Minimum number of occurrences was set to 20. Emulate original motif-x was ticked, and other parameters with default.

### Enrichment-based clustering

For further hierarchical clustering based on differentially modified protein functional classification (such as: GO, Domain, Pathway, Complex). We first collated all the categories obtained after enrichment along with their *P* values, and then filtered for those categories which were at least enriched in one of the clusters with P value < 0.05. This filtered P value matrix was transformed by the function x = −log10 (*P* value). Finally these x values were z-transformed for each functional category. These z scores were then clustered by one-way hierarchical clustering (Euclidean distance, average linkage clustering) in Genesis. Cluster membership were visualized by a heat map using the “heatmap.2” function from the “gplots” R-package.

### Protein-protein interaction network

All differentially expressed modified protein database accession or sequence were searched against the STRING database version 10.5 for protein-protein interactions [18]. Only interactions between the proteins belonging to the searched data set were selected, thereby excluding external candidates. STRING defines a metric called “confidence score” to define interaction confidence; we fetched all interactions that had a confidence score > 0.7 (high confidence). Interaction network form STRING was visualized in R package “networkD3”.

### Western blot

Proteins were run 12% SDS-PAGE gels, then adjust the voltage to the 100 V and electrophoresis for 15 min. After the bromophenol blue migrated out of the concentrated gel, the voltage was switched to 200 V for 40 min. And transferred to a polyvinylidene fluoride (PVDF) membrane by semi-dry technique and set the power to 100 V (constant voltage) for 1 h at 4 °C. The membranes were blocked in TBS (Tris buffered saline) containing 0.05% (v/v) Tween 20 with 5% (w/v) skim milk and incubated 1 h at room temperature. The primary antibodies used in the western blot were anti-Kcr (1:2000), anti-YebC (1:5000), anti-CsbD (1:4000), anti-Sal (1:4000), anti-Coldshock (1:4000) and incubated overnight at 4 °C. After washing in 10 mM PBS (pH 7.4) containing 0.1% Tween-20, the membranes were incubated in horseradish peroxidase (HRP) conjugated goat anti-rat IgG (CWBIO, China) diluted 1:5000 at room temperature for 3 h. Finally, the membrane was visualized using the ECL system (Bio-Rad, Hercules, CA, USA), and recorded by the ChemiDoc™ MP (Bio-Rad, Hercules, CA, USA) imaging system [19].

## Results and discussion

### Detection of lysine-crotonylated proteins in *S. agalactiae*

**Fig. 1:**
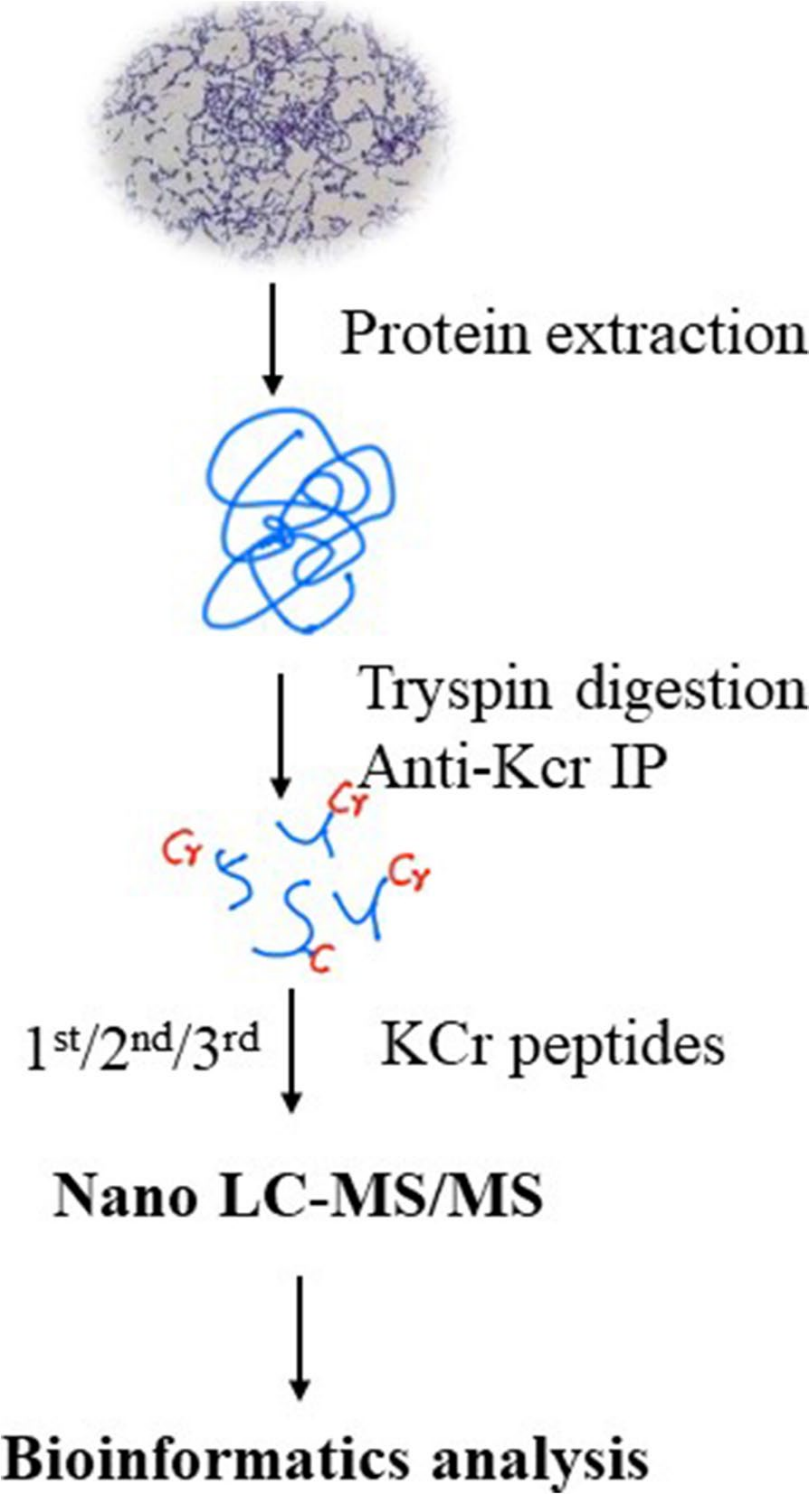
Overview of experimental procedures used in the present study. Kcr indicates the crotonylated lysine

**Fig. 2:**
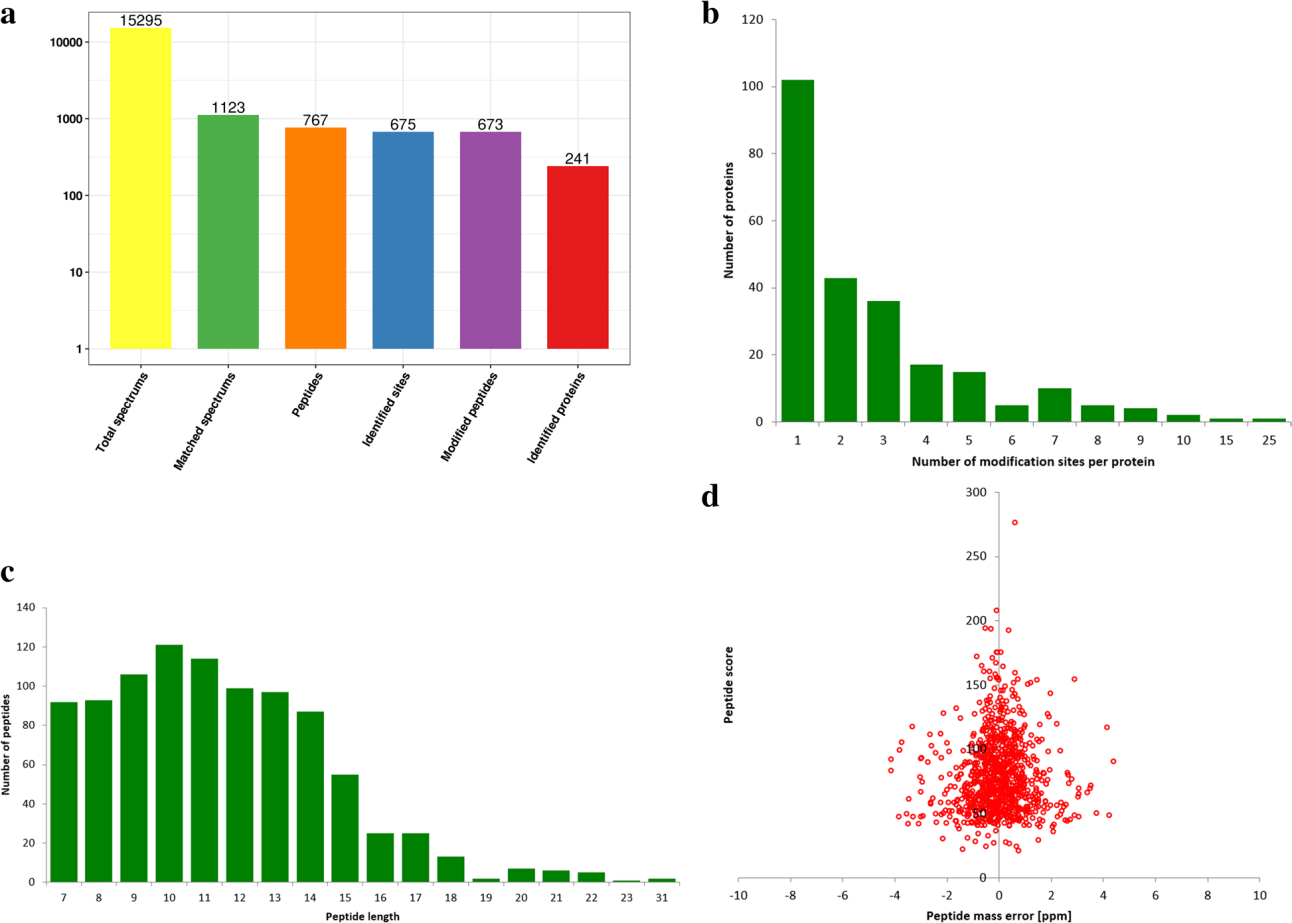
Proteome-wide identification of lysine crotonylation sites in S. agalactiae. **a** Basic statistical figure of MS results. **b** Number of modification sites per protein. **c** Distribution of lysine crotonylation peptides based on their length. **d** Mass error distribution of all crotonylated peptides

To obtain the global crotonylation proteome of *S. agalactiae*, a proteomic method based on sensitive immune-affinity purification and high-resolution LC-MS/MS was applied to identify crotonylated proteins and their modification sites in *S. agalactiae.* An overview of the experimental procedures is shown in Fig. 1. A total of 241 lysine crotonylation sites distributed in 675 proteins were identified. Detailed information for all identified crotonylated peptides and their corresponding proteins was shown in (Fig. 2a, Supplementary Table S1). Among the 675 crotonylated proteins, 145 (21%) proteins contained one or two crotonylation sites, and 13 (1%) proteins had 7 or more crotonylation sites (Fig. 2b). Most peptides ranged from 7 to 15 amino acids in length (Fig. 2c). For quality control validation of the MS data, we evaluated the mass error of all identified peptides. The mass error of most crotonylated peptides ranged from − 5 to 5 ppm, indicating an expected error control from the MS dataset (Fig. 2d).

### Motifs and secondary structures of lysine crotonylated peptides

**Fig. 3:**
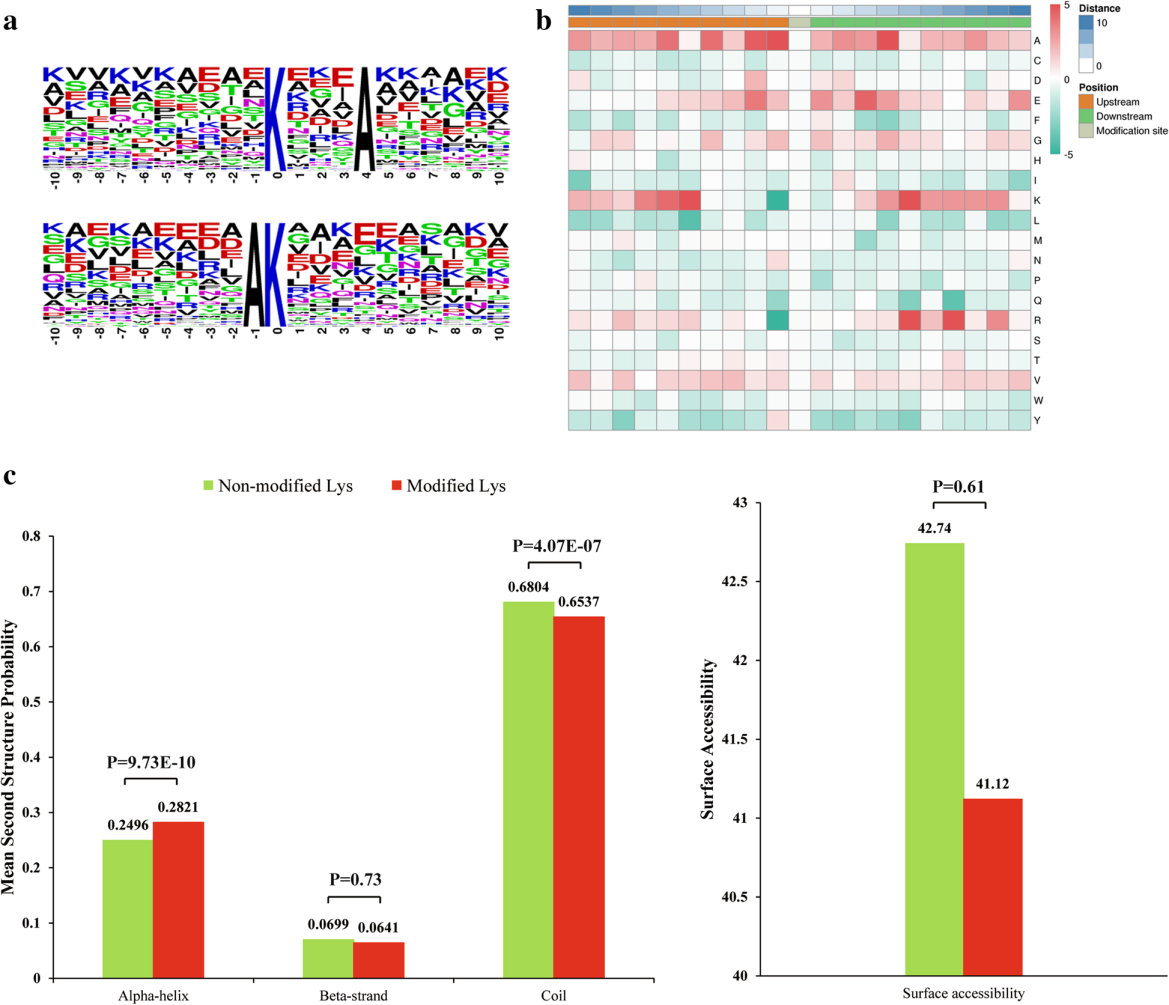
Properties of the lysine crotonylation sites. **a** Sequence probability logos of significantly enriched crotonylation site motifs around the lysine crotonylation sites. **b** The motif enrichment heatmap of upstream and downstream nmino acids of all identified modification sites. Red indicated that this amino acid is significantly enriched near the modification site, and green indicates that this amino acid is significantly reduced near the modification site. **c** Probabilities of lysine crotonylation in different protein secondary structures (alpha helix, beta-strand and disordered coil). **d** Predicted surface accessibility of crotonylation sites

To evaluate the nature of the crotonylated lysines in *S. agalactiae*, the sequence motifis in all identified crotonylated peptides were investigated using the Motif-X programme [20]. Of all surrounding sequences, 164 sequences were matched to a total of two definitively conserved motifs, there motifs were K***A^kcr^, AK^kcr^ shown in Fig. 3a. These motifs are likely to represent a feature of crotonylation in *S. agalactiae*. Hierarchical cluster analysis was also performed to further analyze these motifs. As shown in the heat map (Fig. 3b), the enrichment of negatively charged A residues was observed in the − 10 to − 6, − 4 to + 4 and − 6 to + 10 position, while negatively charged residues E was markedly enriched in the − 6 to + 6 position, the residues K was markedly enriched in the − 10 to − 6 and + 3 to + 9 position.

To explore the relationship between lysine crotonylation and protein secondary structures, a structural analysis of all crotonylated proteins was performed using the algorithm NetSurfP. As shown in Fig. 3c, approximately 28% of the crotonylated sites were located in ɑ-helices, and 7% of the sites were located in β-strands. The remaining 65% of the crotonylated sites were located in disordered coils. The surface accessibility of the crotonylated lysine sites was also evaluated. The results showed that 41.12% of the crotonylated lysine sites were exposed to the protein surface, close to that of all lysine residues (Fig. 3d). Therefore, lysine crotonylation likely does not affect the surface properties of modified proteins.

### Functional annotation and subcellular localization of crotonylated proteins

**Fig. 4:**
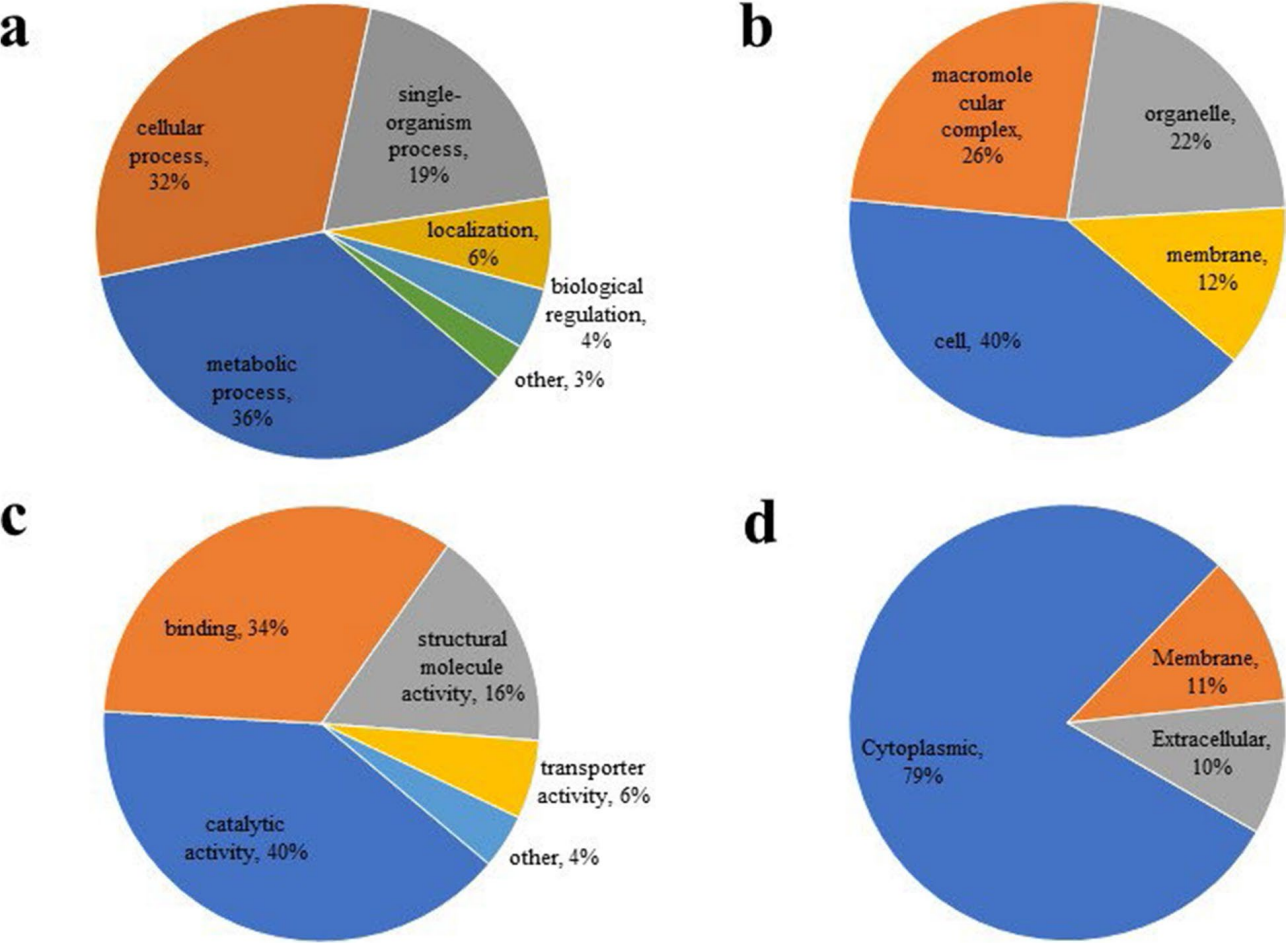
GO classification of the crotonylated proteins based on biology process (**a**), cellular component (**b**), molecular function (**c**) and Subcellular localization (**d**)

To obtain an overview of crotonylated proteins in *S.agalactiae*, the Gene Ontology (GO) functional classification of all crotonylated proteins based on their biological processes, cellular component, molecular function and subcellular locations was investigated (Supplementary Table S2). Within the biological processes category, the majority of crotonylated proteins were associated with metabolic processes, cellular process, and single-organism processes, respectively accounting for 36, 32 and 19% of all the crotonylated proteins (Fig. 4a). For the cellular component category, 40, 26 and 22% of the crotonylated proteins were associated with cell, macromolecular complex and organelle, respectively (Fig. 4b). In the molecular function category, the majority of catalytic activity, binding and structural molecule activity, respectively accounting for 40, 34 and 16% of all the crotonylated proteins (Fig. 4c). Subcellular localization analysis revealed that most of the crotonylated proteins were localized in cytoplasmic (79%), membrane (11%) and extracellular (10%) (Fig. 4d).

### Functional enrichment analysis

**Fig. 5:**
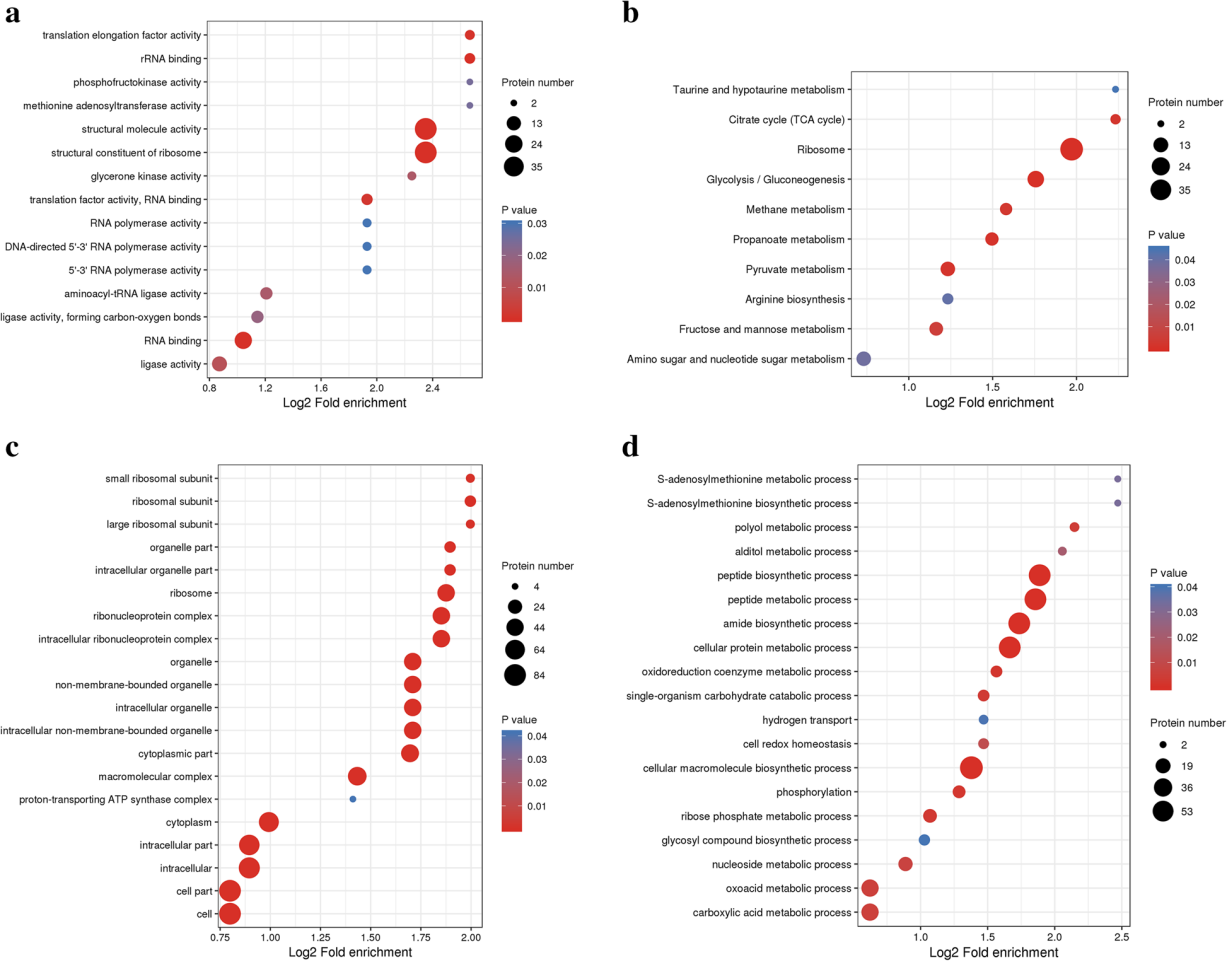
GO (Gene Ontology) and KEGG pathway enrichment bubble plot of proteins corresponding to modification sites. **A** Molecular Function **B** KEGG pathway enrichment bubble plot **C** Cellular Component **D** Biological Process

To better comprehend the biological function of these crotonylated proteins, we performed an enrichment analysis of the GO (Supplementary Table S3), Kyoto Encyclopedia of Genes and Genomes (KEGG) pathway (Supplementary Table S4), and Pfam domain databases (Supplementary S5). The enrichment analysis of the biological process revealed that the crotonylated proteins were significantly enriched in the peptide biosynthetic process, peptide metabolic process, amide biosynthetic process, cellular protein metabolic process and cellular macromolecule biosynthetic process (Fig. 5a). Based on the enrichment results of the cellular component category, most crotonylated proteins were related to cell, cell part, intracellular and intracellular part (Fig. 5b). In the molecular function category, structural molecule activity, glycerone kinase activity and RNA binding (Fig. 5c). The KEGG pathway enrichment analysis showed that a majority of the crotonylated proteins were ribosome-related proteins, Glycolysis/Gluconeogenesis, methane metabolism, pyruvate metabolism, propanoate metabolism, citrate cycle (TCA cycle) and fructose and mannose metabolism (Fig. 5d, Supplement Figs. 1, 2, 3, 4, 5, 6, 7, 8, 9 and 10). Consistent with these observations, Pfam domains, including the Aldehyde/histidinol dehydrogenase, Aldehyde dehudrogenase (C-terminal and N-terminal), Aldehyde dehydrogenase domain and translation protein (beta-barrel domain) (Fig. 5d), were significantly found in crotonylated proteins, implying an important role for lysine crotonylation in these processes.

### Protein interaction network of the crotonylated proteins in *S. agalactiae*

**Fig. 6:**
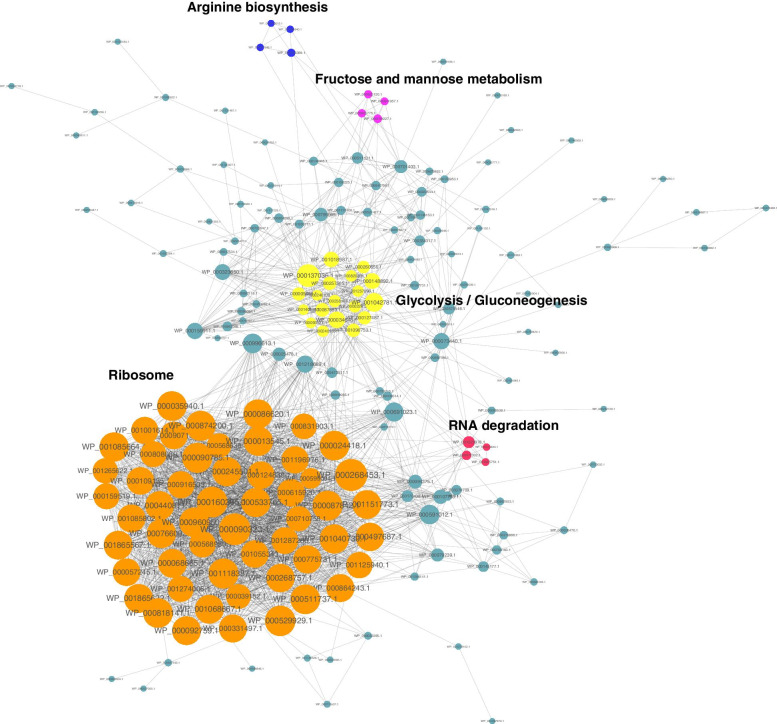
Interaction networks of the crotonylated proteins in *S. agalactiae*.

To further identify the cellular processes regulated through crotonylation in *S. agalactiae*, the crotonylated protein interaction network was established using an algorithm in Cytoscape software. A total of 193 crotonylated proteins were mapped to the protein interaction database (Supplementary Table S4). As shown in Fig. 6, ribosomal, arginine biosynthesis, fructose and mannose metabolism, Glycolysis/Gluconeogenesis and RNA degradation proteins were highly interconnected, indicating that these translation process may be regulated by crotonylated in *S. agalactiae*. The physiological interactions among these crotonylated protein complexes may contribute to their cooperation and coordination in *S. agalactiae*. The results of PPI networks suggest that lysine crotonylation probably play a role in regulating post-translational modification of protein in *S. agalactiae,* which contributes to cooperation and coordination of metabolic pathways.

### Validation of ten lysine-crotonylated proteins using Western blotting

**Fig. 7:**
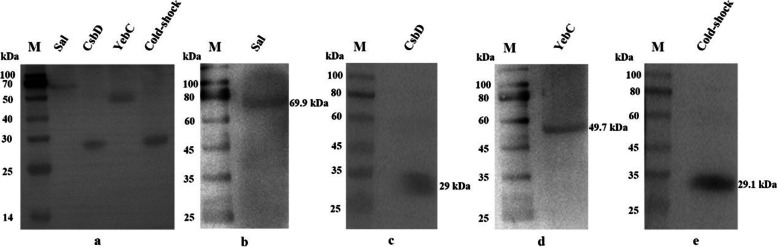
**a** Validation of Sal(69.9kDa), CsbD (29kDa),YebC (49.7kDa), and cold shock protein (29.1kDa) lysine-crotonylation proteins in *S. agalactiae* using Western blotting. Twenty μg of recombinant expressed protein were loaded, and crotonylated proteins were detected with anti-Kcr monoclonal antibody (1:2000). **b** Sal (69.9kDa) protein in *S. agalactiae* using Western blotting with specific antibody (1:2000). **c** CsbD (29kDa) protein in *S. agalactiae* using Western blotting with specific antibody (1:2000). **d**YebC (49.7kDa) protein in *S. agalactiae* using Western blotting with specific antibody (1:3000). **e** cold shock protein (29.1kDa) in *S. agalactiae* using Western blotting with specific antibody (1:2000)

To further validate the identified lysine-crotonylated results, four Kcr proteins (YebC/PmpR family DNA-binding transcriptional regulator [21], surface antigen-like protein [22], cold-shock protein [23], diacylglycerol kinase, CsbD [24]) were selected and analyzed by western blotting. The four proteins were identified with anti-crotonylation and anti-target protein antibody, respectively (Fig. 7). Consistent with lysine-crotonylated proteomic data, the all four detected proteins exhibited crotonylation modifications.

## Conclusion

In this study, using high-resolution LC-MS/MS coupled with highly sensitive immune-affinity purificationwe, we investigated the global lysine crotonylation proteome of *S. agalactiae*. We reported the proteome-wide profiling of Kcr in *S. agalactiae* and identified 241 Kcr sites of 675 proteins for the first time. The bio-informatics analysis showed that 164 sequences were matched to a total of six definitively conserved motifs, and many of them were enriched in metabolic processes, cellular process, and single-organism processes. Moreover, we also found a considerable four crotonylation modified proteins involve in sensing system and bacterial virulence, potentially providing targets for vaccine development. The present data provides new insights into the pathogenesis of *S. agalactiae* from fish, and the virulence mechanisms should be further investigated.

## Supplementary Information

**Additional file 1.**

**Additional file 2.**

## Data Availability

All data generated or analyzed during this study are included in this published article and its supplementary information files.
