# Supplementary material for: First comprehensive proteome analysis of lysine crotonylation in Streptococcus agalactiae, a pathogen causing meningoencephalitis in teleosts
Source: Proteome Sci. 2021 Nov 10;19:14. doi: 10.1186/s12953-021-00182-y (PMC8580364; doi:10.1186/s12953-021-00182-y)

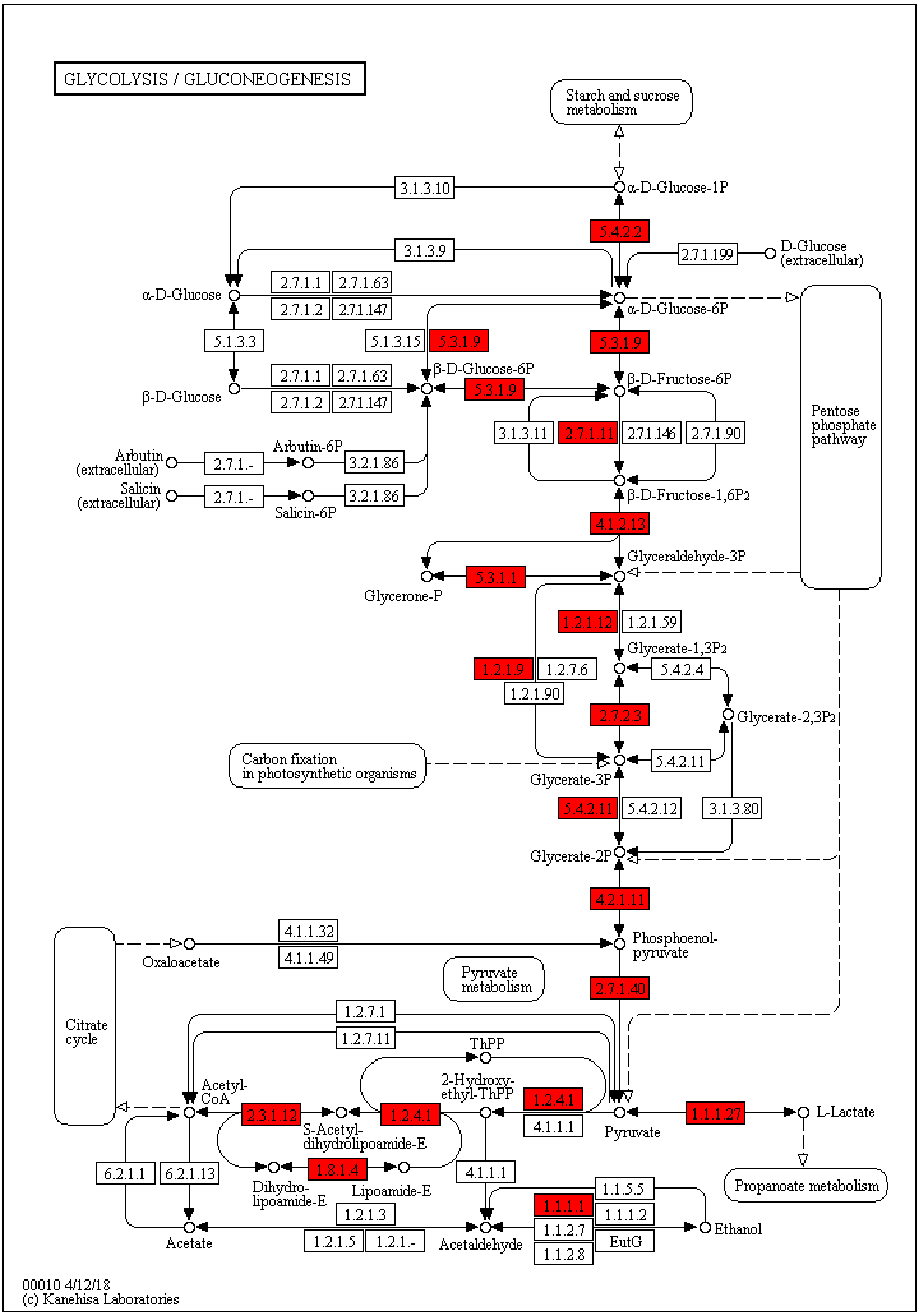

Supplementary Figure 1

Supplementary Figure 2

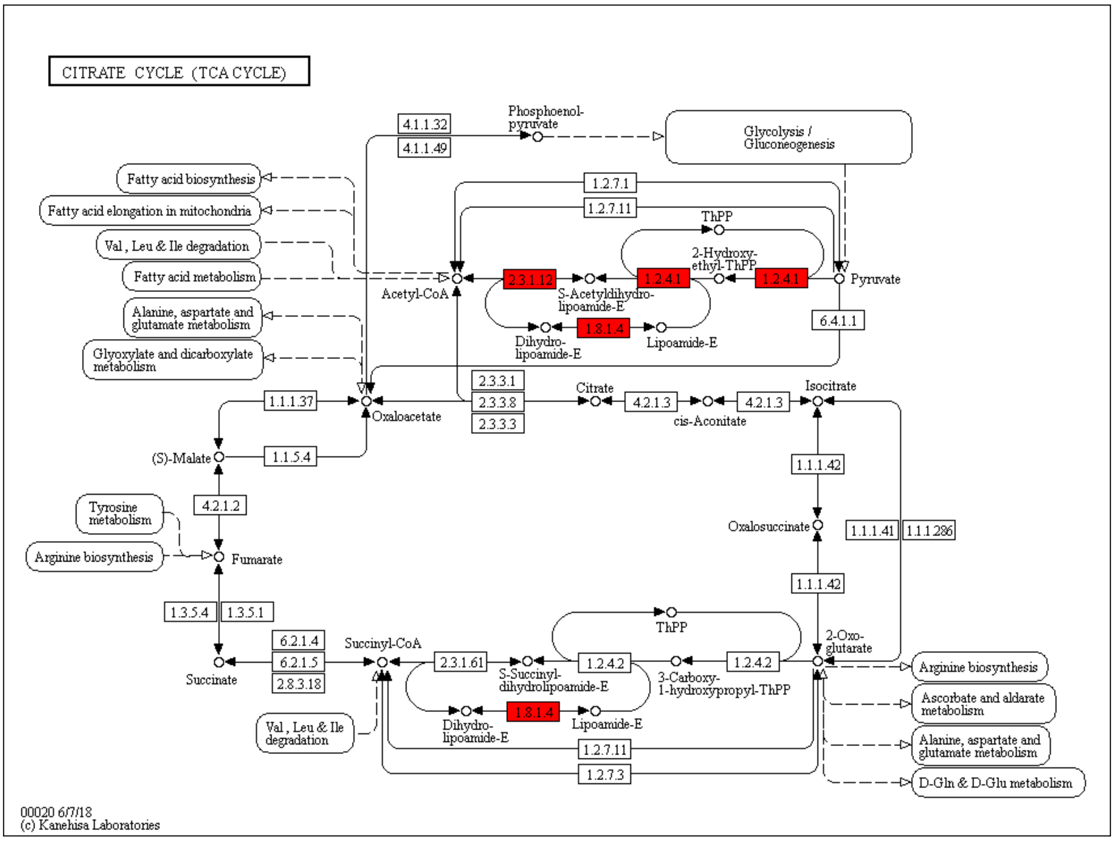

Supplementary Figure 3

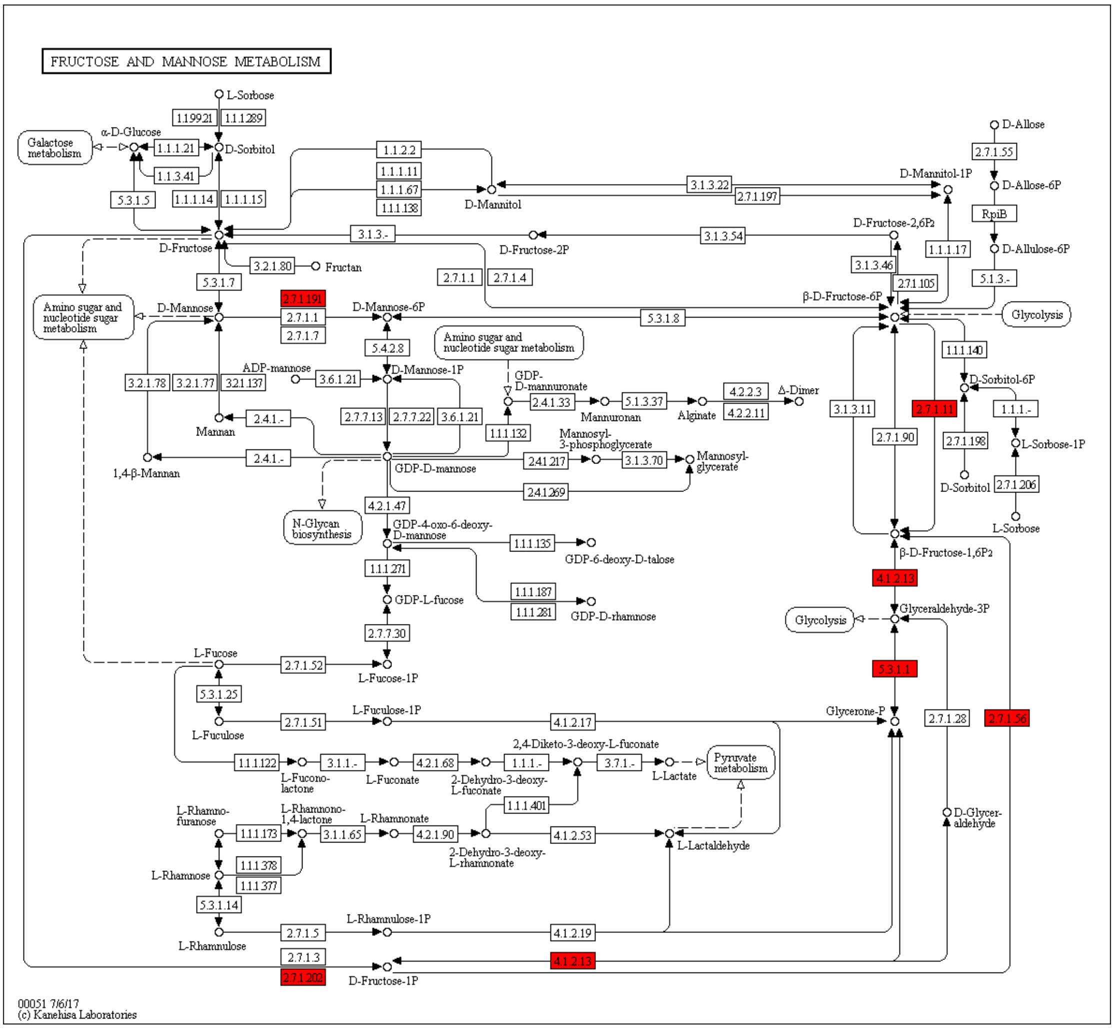

Supplementary Figure 4

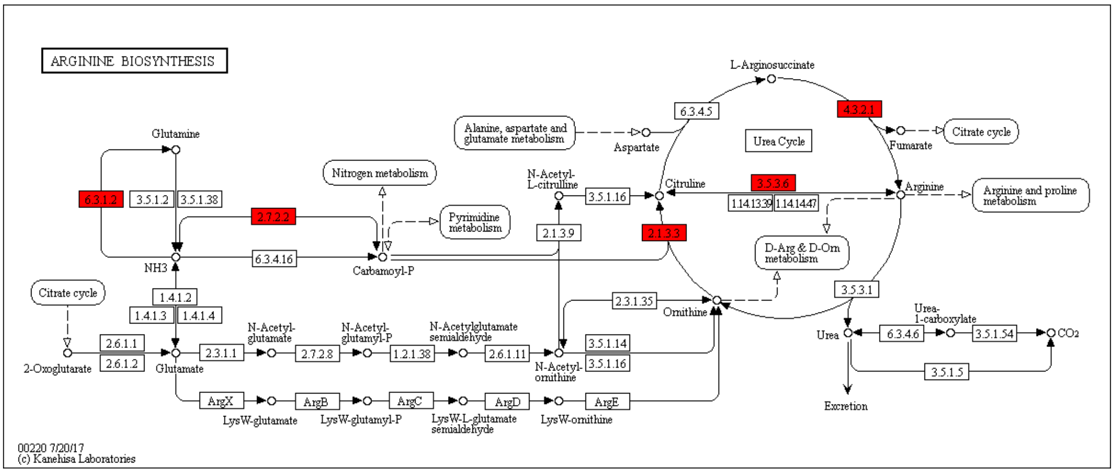

Supplementary Figure 5

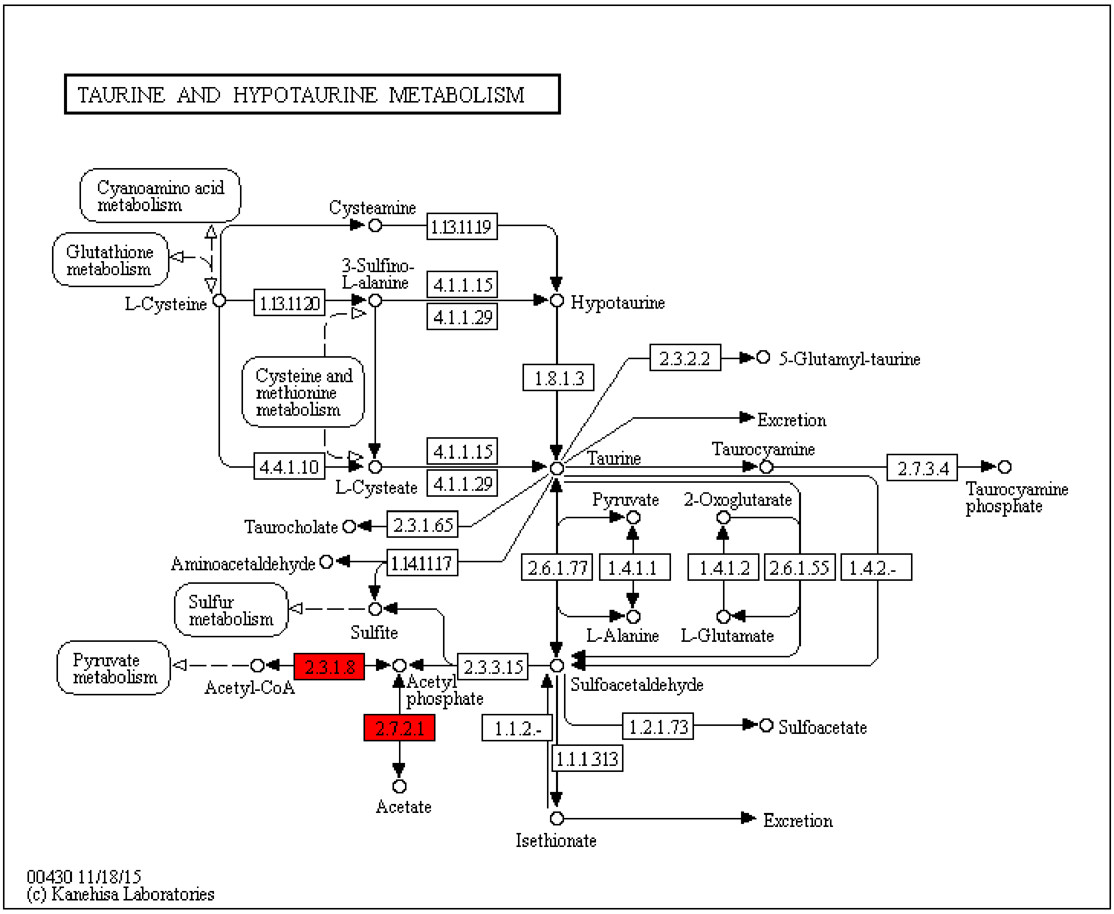

Supplementary Figure 6

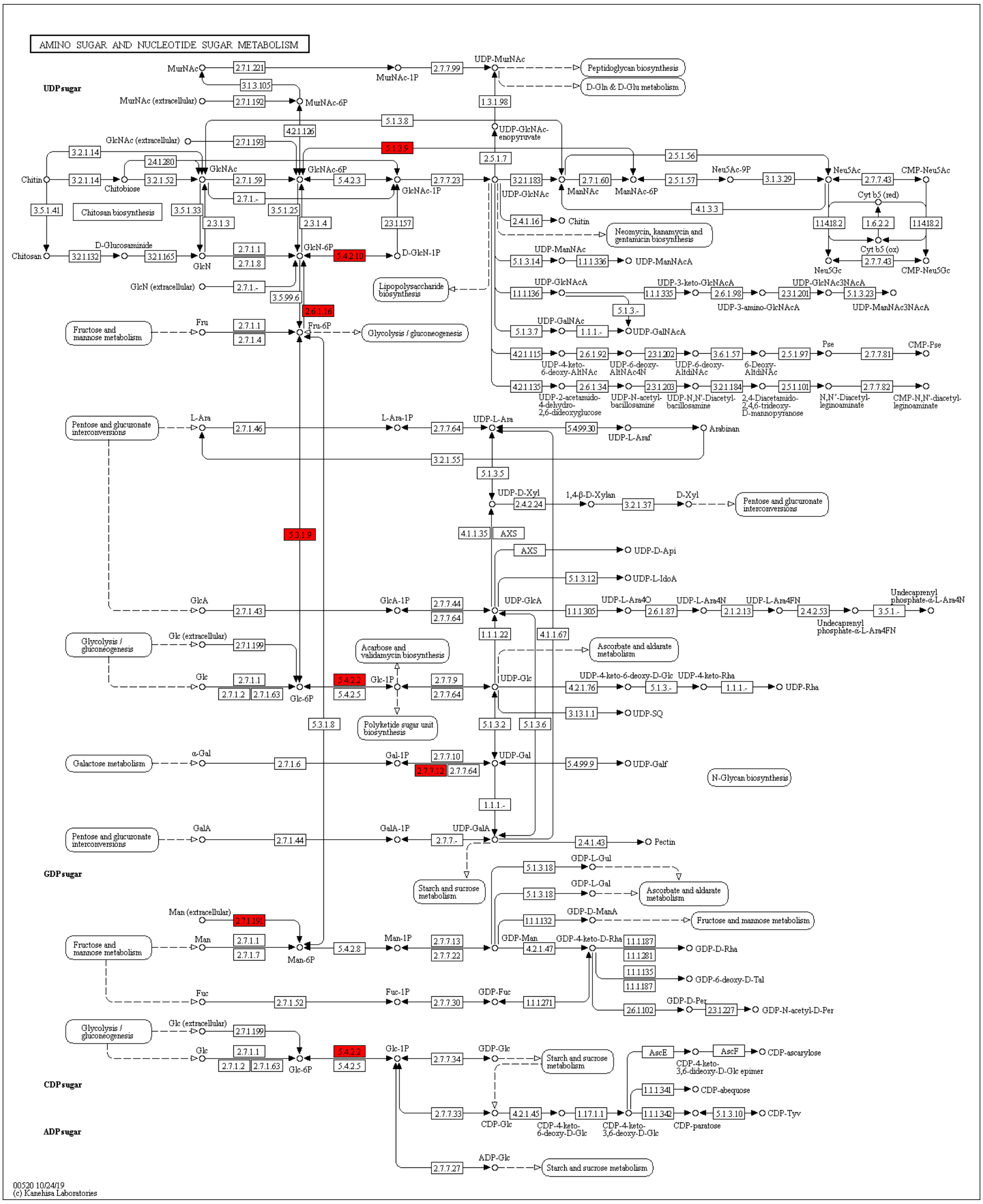

Supplementary Figure 7

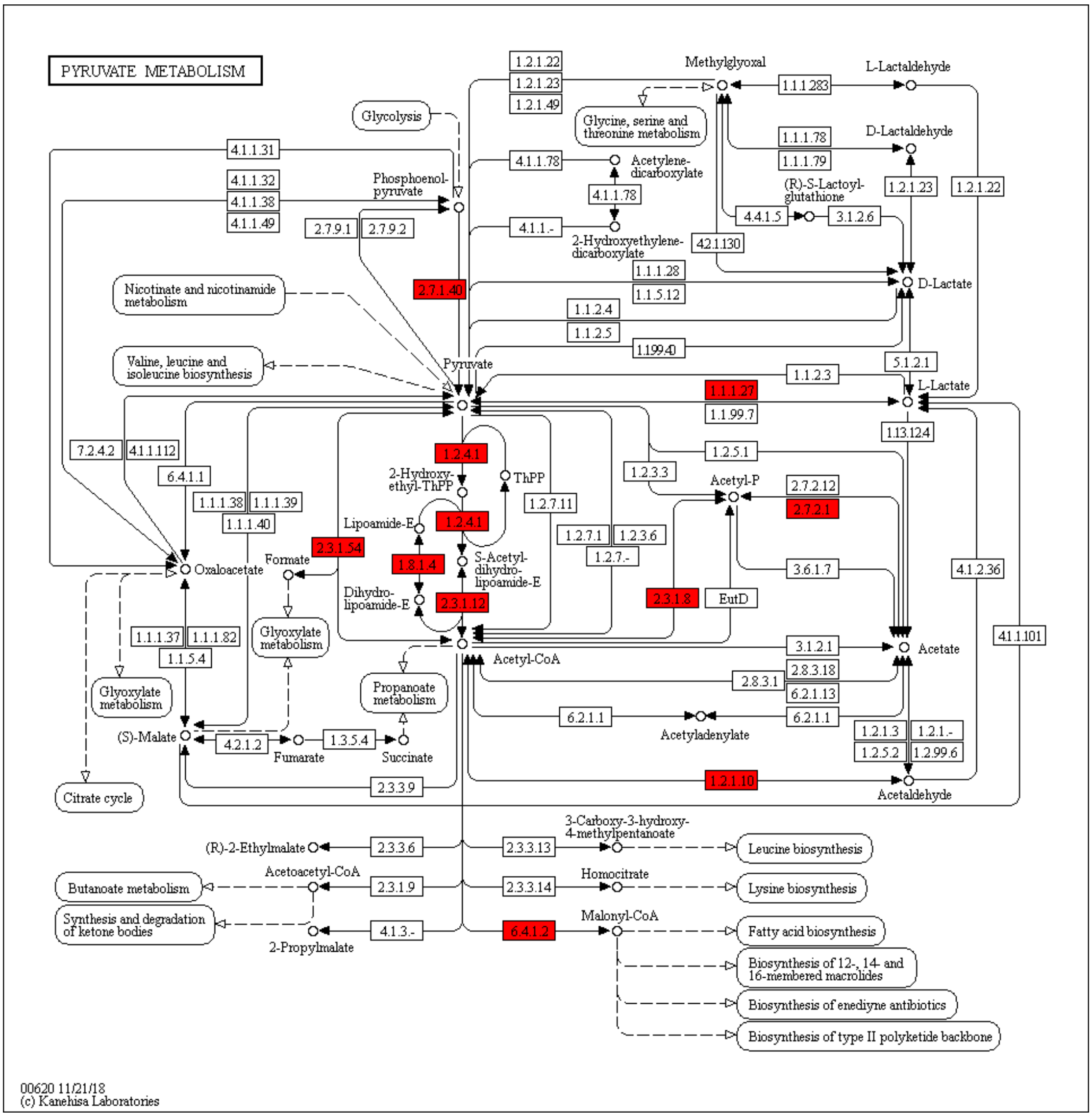

Supplementary Figure 8

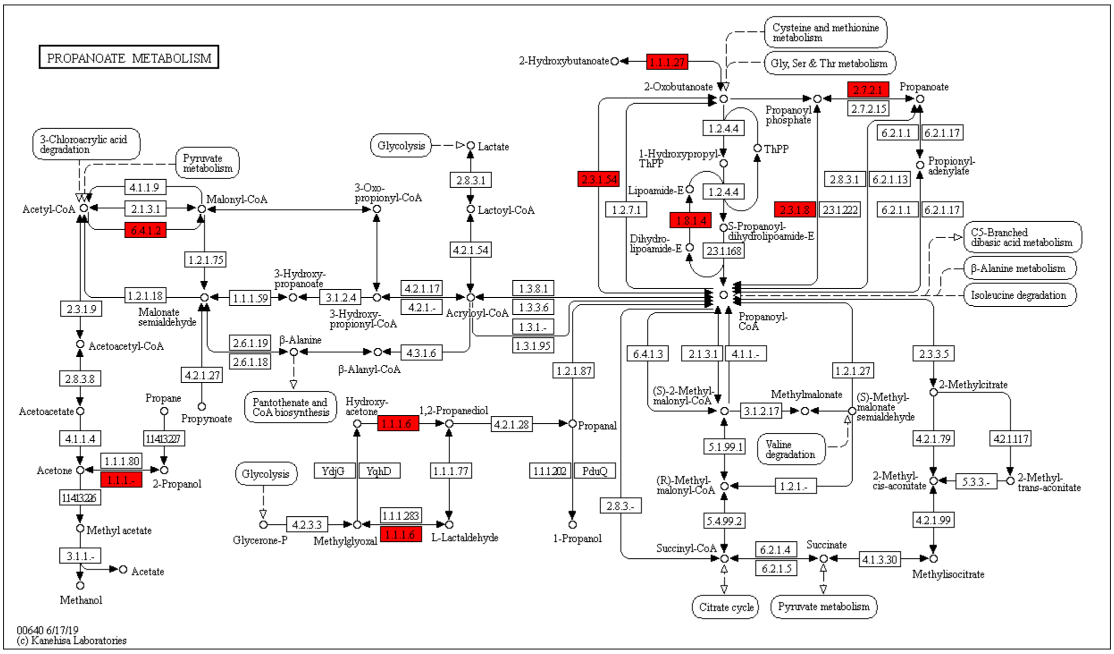

Supplementary Figure 9

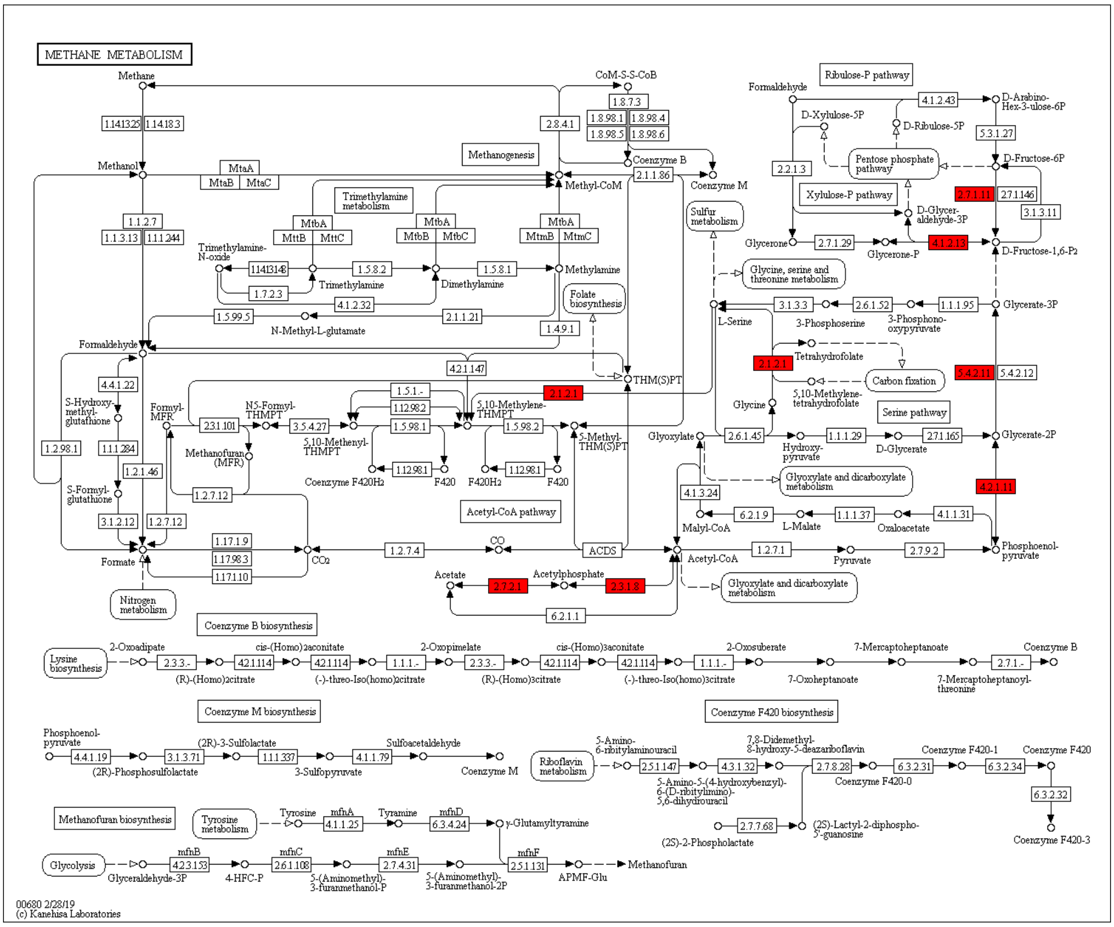

Supplementary Figure 10

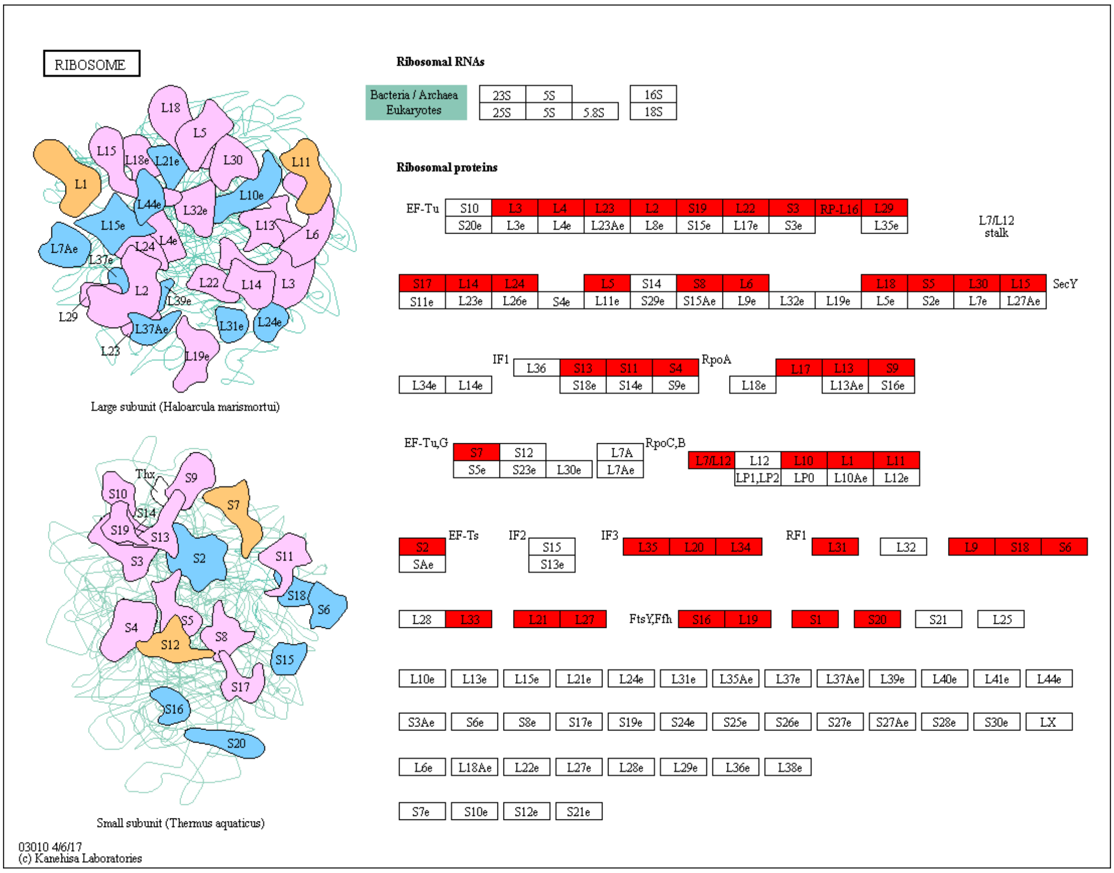

Supplement: Supplementary file 2 — Additional file 2. [file 12953_2021_182_MOESM2_ESM.pdf]
